# Determinants of Gender Disparity in Nutritional Intake among Children in Pakistan: Evidence from PDHS

**DOI:** 10.3390/children9010007

**Published:** 2021-12-24

**Authors:** Awaisra Shafiq, Abid Hussain, Muhammad Asif, Arif Jameel, Saiqa Sadiq, Shahida Kanwel

**Affiliations:** 1 Department of Economics, The Islamia University, Bagdad ul Jadeed Campus, Bahawalpur 63100, Pakistan; awaisra017@gmail.com; 2 School of Management, Jiangsu University, Zhenjiang 212013, China; abidhusssain02@gmail.com (A.H.); arifjamil24@gmail.com (A.J.); 3 School of Governance and Society, University of Management and Technology, Lahore 54770, Pakistan; 4 School of Public Affairs, Zhejiang University, Zijingang Campus, Hangzhou 310058, China; 5 College of Law, Government & International Studies, University Utara Malaysia, Bukit Kayu Hitam 06010, Malaysia; drsaiqasadiq@gmail.com; 6 Hotel & Tourism Management, School of Management, Zhejiang University, Hangzhou 310058, China; shahidakanwel@yahoo.com

**Keywords:** PDHS, malnutrition, wealth index, gender disparity, national nutrition survey, children

## Abstract

The purpose of this study is to analyze early age malnutrition on a gender basis in Pakistan. Pakistan Demographic and Health Survey (PDHS) 2012–2013 data related to households’ characteristics that affect the nutrition of children less than 5 years of age are used for the estimation of results. Gender disparity (measured by girl malnourished in household/boy malnourished in a household) is constructed for the measurement of gender disparity in early age child nutrition. After synthesizing the PDHS data set, 2119 observations are used for regression results of gender disparity. Regression results are analyzed at the level of 5% confidence interval otherwise insignificant. egression results for gender disparity show that households in good socioeconomic status, a greater number of household members, a mother’s higher level of education, mother employment, and the male head of the household, causes a decrease in gender disparity in nutrition intake of children.

## 1. Introduction

Child health is a key indicator of economic development. In most developing countries, nutrition continues to be a major public concern. The main determinants of child malnutrition are poverty, population growth, unawareness, lack of excess food, the burden of diseases, illiteracy, environmental damage, i.e., lack of safe drinking water, and inequality among children. Gul and Kibria [1] exposed that child’s malnutrition is directly linked to low family income, mother’s illiteracy, and large family size that causes higher mortality and morbidity rate. In developing countries, children from the poorest houses are twice as malnourished than children from the richest houses [2].

Among female children, a major cause of malnutrition is gender disparity, gender inequality in quantity and quality of food intake mainly due to the reason that a female child is considered less important than a male child. In several parts of South Asia, gender inequality in nutrition, education, and health is reported consistently [3]. Matanda, et al. [4] found that if both girl and boy children under 5 years of age are cared for and nourished in the same way, then their anthropometric status could be similar. The difference in diet is attributed to the difference in long-term physical development.

Gender and nutrition are an inextricable part of a vicious cycle of poverty. Sex bias is the most common phenomenon in poor houses. In a poor society, wrong, internalized norms exist among parents that lead to better care for their sons as compared to their daughters [5]. In a poor household, mothers give food to their sons and husband first and later give it to their daughters and eat them. Most probably, insufficient and poor quality food is left for females [6]. In poor houses, sons are more preferred than daughters since it is believed that sons would be their source of income in their old age, but the daughter would be a burden. So this concept of parents and elders of households creates discrimination in the allocation of food, clothing, education, and medical expenses [5]. In Pakistan, poverty is higher in rural and poor urban areas. Women and girls are more discriminated against more than health outcomes and nutritional intake [7].

## 2. Literature Review

Healthy nutrition for children is necessary for a strong immune system, cognitive development, healthy growth, and proper organ formation. In many societies, women and girls faced the situation of food discrimination, which in turn causes chronic undernutrition and ill health [5]. Risk factors in Bangladesh were identified among children that cause diarrhea and also gender differential between these hospitalized children that causes death. Girls were facing higher numbers of diarrheal infections due to higher body temperature, incomplete or no immunization, malnutrition, and electrolyte imbalances, etc. Mitra, et al. [8] and Choudhury, et al. [9] revealed that girls are more severely malnourished as compared to boys. Children who could not receive DPTI were less malnourished as compared to others who received it.

A study on rural Eastern Kenya analyzed that girls of all ages are more malnourished (stunted, wasted, and underweight) as compared to boys due to lower food intake [4]. Mother’s age and education were significantly associated with severe malnutrition. Mother’s education positively affects child nutritional outcomes in Bangladesh [9]. In Mozambique, a rural–urban comparison between two states of India Bihar and Jharkhand determined that despite a mother’s education, no difference exists among child nutrition whether the mother belongs to a rural or urban area. Results also determined that there exists significant nutritional discrimination between male and female children [10]

The good socioeconomic status of households helped to reduce the gender disparity in nutritional outcomes. Malnutrition is not only a symptom of ill health, but also a cause of poor socio-economic status [6]. Gender differential in the treatment of girls’ and boys’ sickness at household level causes to higher mortality rate and risk of malnutrition in female children [11]. In Malawi, Tanzania, and Zimbabwe, a mother’s education affects more beneficially male child nutrition than a female child. Similarly, mother employment is also negatively related to gender disparity in their nutritional and health outcomes [12]. Brazil’s study determined that when the head of the household is a woman, and all the income-expenditure is controlled by women, then the probability of children to survive with good health increases [13,14].

The research gap determines that no single study is found that uses the data of PDHS (2012–2013) for the gender disparity of malnutrition on a gender basis. However, the current study is organized to fill this research gap to some extent.

## 3. Theoretical Framework

The etiology of malnutrition is difficult to understand. Poor nutritional status of children is due to a combination of several social, economic, psychological, and environmental, and health factors. The conceptual framework that we use to examine the nutritional status of children is based on the utility maximization model of households. In the modeling of child’s nutritional determinants, we have assumed that each child lived in a unit called a household; these households maximize utility functions as follows:

*U* = *u* (*H^i^*, *F^i^*, *Z^i^*, *L^i^*) (1)

The model shows that a household’s utility depends on the nutritional health (*H^i^*) of individuals, consumption of food and nonfood expenditures (*F^i^*, *Z^i^*), and leisure (*L^i^*) of individuals. All these factors are defined in several dimensions [15].

## 4. Materials and Methods

For the empirical analysis, data is taken from PDHS (Pakistan Demographic and Health Survey 2012–2013). It is the third survey organized on a national scale under the supervision of GDHSP (Global Demographic and Health Survey Program). For the analysis of gender disparity (Female malnourished/Male malnourished), 2119 observations are used. These observations are taken from those households that have both male and female children.

This model is used to measure the disparity between the male and female children in the attainment of nutritional values between female and male children. The ratio is one of the methods to construct differential in nutritional status of children. In this method, the dependent variable is converted into the girl over boy ratio (girl/boy) and ensured that gender disparity is captured between the female and male child attainment level under age 5 rather than the level themselves [16].

The gender Disparity model (to measure gender differential) is specified as:

GD (girl malnourished in a household/boy malnourished in a household) = f (MEDU_ij_, WI_ij_, NCH_ij_, HHS_ij_, REGION_ij_, PRES_ij_, MEMPL_ij_, HHead_ij_) (2)

The regression model for gender disparity analysis is,

GD = β_0_ + β_1_MEDU + β_2_WI + β_3_NCH +β_4_HHS +β_5_PRES +β_6_MEMPL +β_7_HHEAD +ε_0_ (3)

The dependent variable, Gender Disparity (GD) is used as a measure of the disparity in the nutritional status of less than 5 years of age children (Table 1). It is measured by the ratio between the malnourished girl and boy child (girl malnourished/boy malnourished). This method is measured and analyzed by using the concept given in the global gender report. The Global Gender Gap Report examines the gap between male and female children by using an index that is called Global Gender Gap Index. The four fundamental categories are used to measure this gap. Health and survival are one of the major categories [16].

To construct Gender Disparity, all households selected are those where both male and female child whose age is under 5 and they are stunted, wasted, or underweight. In this process three conditions are kept in mind.

Subsets of those households are taken in where stunted, wasted or underweight male and female child are taken into consideration. For example, if a girl child is stunted while a boy child is wasted then we consider both malnourished and find the ratio.

Subsets of those households are not taken into consideration where only a male or female child exists. In other words, the household selected to measure disparity in their nutritional status contained both female and male child.

In the final step, the ratio is truncated at the equality benchmark. The equality benchmark is considered 1. This means an equal number of female and male malnourished children. If values of ratio exist below 0 e.g., 0.5, it means the male child is more malnourished than girl child; similarly, if the value of ratio exists greater than 1 e.g., 1.5, it means the female child is more malnourished in a house compared to the boy child.

The regression model is used as a statistical technique for the empirical analysis of results. In this model, independent variables are used in the form of categorical/qualitative/dummy nature, since the purpose is to measure the gender disparity in the nutritional status of children. Two possible scales are used for the interpretation of coefficients to capture gender disparity. One is a positive-negative scale that is used to capture the size and direction of the gender gap in nutrition as it is increasing or decreasing. This scale helps to determine that in a house whether the male child has an advantage over the female child, or the female child has an advantage over the male child. Another scale is named as a one-sided scale that helps to determine that how close female children are reaching parity with male children [16].

## 5. Results

For better results, we used descriptive statistics (frequency, mean, and standard deviation) of variables in our study [17]. Frequencies explain the distribution of each variable (Gender Disparity, Mother Education, Wealth Index, Number of Children in a house, Household Head, Household Size, Place of Residence, and Mother Employment) according to their categories (Table 2).

To measure disparity between male and female children, all the household and maternal characteristics are used that are important to create differential. A ratio that is measured by “girl malnourished/boy malnourished” is used as a dependent variable to capture the effect of all other factors (independent variable) on Gender Disparity. After synthesizing the PDHS total number of observations used in the analysis are 2119. Statistical software SPSS and STATA are used to measure the results by using the Regression technique [18,19]. Few variables exist in the categorical form and data exist in cross-sectional values of R^2^, for the overall significance of the model is not important but the individual significance “*p*-value” of variables is important for analysis [20]. Results show that variables are significant because the t-values are greater than a cutoff point value of 1.96 [21,22] (Table 3).

## 6. Discussion

Results show that an increase in wealth index (poorest to richest) causes a reduction in gender disparity. According to a study in India, the good socioeconomic status of households helped to reduce the gender disparity in nutritional outcomes. Malnutrition is not only a symptom of ill health, but also a cause of poverty [18].

In developing countries, gender bias is a common phenomenon. The female child did not share and care optimally as a male child. The reason for higher malnutrition in children is not only lack of nutritious food and poverty but also value attached to the girls in their families from early adult age. In poor houses, children are more malnourished since they have less or no money to purchase food. This is the reason mothers’ employment in poor houses causes them to improve the nutrition of their children [23]. A similar study was founded in rural Punjab of India to measure selective discrimination against female children. The results of this study showed that landless (linked to poor socioeconomic status) households discriminated more against girls for expenditures of food, clothing, and medicine as compared to land-owning households (indicated good socioeconomic status) [5,24].

Another important variable that causes an increase in gender differential between male and female children is the number of children under age 5 in the household. The results show that the increase in the number of children under age 0–5 causes to increase in gender disparity. If in a house where the number of children under age five is more, the female child of that house is more malnourished as compared to the male child. This study is similar to the study of Mozambique, which found that increase in household members from age 0–5 causes to increase in height for age Z-score [25]. In North Shewa, the Regional State of Oromia number of children under 5 in a house is associated significantly with being underweight. Families having three children below five years of age were 4.5 times more likely to be underweight as compared to families having only one child below age five [23].

Similarly, an increase in the number of household members causes a decrease in gender disparity. The negative sign of the coefficient in regression results indicates that an increase in some household members causes a decrease in the gender gap between female children and male children in their nutritional outcomes. It can be due to an increase in the caring person of children and an increase in earned persons. It is supported by a study Al-Hussein and Kano [26], who concluded with their results that in smaller families of urban areas, the risk of malnutrition is higher [25,27].

Results of a mother’s education are significant according to the theoretical background. The findings of previous studies supported that a higher level of maternal education reduced the odds of stunting in Zimbabwe, Tanzania, and Malawi. The mother’s education affects the male child’s nutrition more beneficially than the female child’s [12,28].

Similarly, a mother education’s and its status in society is also an important factor that affects the nutritional outcome of children in their adult age. It not only helps to improve the health of the child, but also causes to decrease gender disparity in nutritional and health outcomes [29].

Several factors are associated with malnutrition that differ from one another and also differ among regions; these factors include education, nutritional status of parents, and income [30]. In the analysis of all-India samples of rural households, the mother’s employment is also negatively related to gender disparity in their nutritional and health outcomes. A result of a study concluded that the survival chances of girls comparatively increased due to an increase in the employment opportunities of adult women [31]. In Ethiopia, the impact of maternal employment on child nutrition is positive for children with low nutrition and negative for children with higher nutritional status and reduced gender disparity in nutritional intake in the shorter term. In poor houses, the mother’s engagement in untrained labor work positively affected child health in the short-term [32].

The head of the household being either male or female plays an important role in the provision of food and generation of inequality among children. In this study, regression results for the head of household determine that if the head of the household in Pakistan is male, then it causes a decrease in disparity in nutritional intake (female malnourished in household/male malnourished in the household). Similarly, in Pakistan, if the head of the household is male then there would be 0.171 less chance that child (both male and female) would be unhealthy [20].

## 7. Conclusions

This study has been able to determine that childhood malnutrition remains very high in girls. Malnutrition in girls in their adult age or reproductive age can cause a vicious cycle of undernutrition and poverty. For this purpose, several predictors related to socio-economic status, maternal, and household-related factors are used for empirical analysis. Major causes of gender disparity in nutritional status among early 5 years of age children are due to lack of mother education, poor socioeconomic status, and a greater number of children in a house. Results also determine that a mother’s employment is negatively related to gender disparity in nutritional intake. Therefore, a mother’s education is crucial for child health and nutrition. Special programs and policies should have a consideration about female education.

In summary, the improvement of the nutritional status of children would help avert child deaths from different diseases such as diarrhea, pneumonia, malaria, etc. Consequently, it would reduce neonatal mortality, and the main aim is to reduce poverty and hunger. There is an urgent need for national policies to improve the delivery of education to the people and implement agricultural policies to reduce the poverty level. In other words, improving maternal and child nutrition is a prerequisite for achieving the goal of low mortality. Also, nutritional programs and policies that will try to reduce female illiteracy and provide basic infrastructures in rural areas to reduce gaps in health care between socio-economic groups are likely to succeed. The majority of the poorest household lives in rural areas and the poorest children are more exposed to the risk of being malnourished. Hence, there is an urgent need to build programs that aim to reduce poverty in both rural and urban areas, and which will consider inequalities observed between children.

## Figures and Tables

**Table 1:** Functional Features of Variables.

Variables Name	Interpretation	Functional Definition
	Dependent Variable	
GD (Gender Disparity)	Indicator used to determine the gender disparity among male/female children (girl malnourished/boy malnourished)	It is used as a continuous variable
	Independent Variables	
MEDU (Mother’s Education)	Maternal literacy is a leading indicator of the nutritional knowledge of children.	0 = No education, 1 = primary, 2 = secondary, 3 = higher
WI (Wealth Index)	The indicator is used to determine the socioeconomic status of the household.	1 for poorest, 2 for poor, 3 for the middle class, 4 for rich, 5 for richest.
NCH (Number of children under 5)	The higher number of children in households can cause poor nutrition and help to generate gender discrimination.	number of children is used as a continuous variable
HHS (Household Size)	In the PDHS survey average, HHS has observed 6.8 persons.	HHS is used as a continuous variable.
PRES (type of place of residence)	The indicator determines the rural-urban differential.	1 = urban, 2 = Rural
MEMPL (Mother’s Employment)	The indicator used to determine the effect of mother status on gender health outcomes	1 = Yes,0 = No
H Head (Household Head)	The indicator was used to measure the effect of nutrition differential on a male and female child in the presence of the head of household either male or female.	0 = if female, 1 = if male

**Table 2:** Descriptive statistics of Variables.

Variables	Category	Frequency	Mean	Standard Deviation
Gender Disparity (GD)	No Disparity	977	0.08	0.31
	Boy child are more malnourished than a girl	792	0.07	0.29
	Girl child are more malnourished than a boy	350	0.04	0.11
Mother’s Education	No Education	6722	0.57	0.49
	Primary Education	1687	0.14	0.35
	Secondary Education	2077	0.17	0.38
	Higher Education	1277	0.11	0.31
Wealth Index (WI)	Poorest	2758	0.24	0.42
	Poorer	2359		0.40
	Middle Income	2270	0.19	0.39
	Richer	2196	0.19	0.38
	Richest	2180	0.18	0.39
Number of children under 5(NCH)		11,736	2.43	1.53
Household Head (H Head)	Women	845	0.11	0.24
	Men	10,918	2.83	1.51
Household Size	Continuous	11,736	9.64	5.27
Place of Residence	Rural	6793	0.61	0.53
	Urban	4970	0.43	0.48
Mother Employment	Yes	9483	0.19	0.39
	No	2233	0.80	0.39

**Table 3:** Dummy Regression for Gender Disparity (GD).

Co-variable	Coefficient (βi)	Std. Error	t-Value	*p*-Value
	1. Wealth index (poorest, reference category)			
Poorer	0.018	0.039	−0.5	0.61
Middle	0.02	0.044	0.46	0.09
Richer	−0.012	0.049	−0.25	0.08
Richest	−0.154	0.062	−2.48	0.01 **
	2. Number of children under 5 age in household (continuous variable)			
	0.145	0.01	13.91	0.000 *
	3. Number of Household member (continuous variable)			
	−0.027	0.003	−8.86	0.000 *
	4. Mother Education (no education, reference category)			
Primary	0.035	0.036	0.96	0.33
Secondary	0.098	0.039	2.52	0.01 **
Higher	−0.065	0.053	−1.24	0.11
	5. Mother Employment (not employed, reference category)			
Employed	−0.088	0.031	−2.81	0.001 ***
	6. Place of residence (Rural, reference category)			
Urban	0.034	0.032	1.03	0.31
	7. Head of the household (female, reference category)			
Male	0.115	0.05	2.29	0.01 **
Intercept (c)	0.323	0.062	5.25	0.001 ***
Model		Overall Effect		
Number of observations		2901		
Prob > F		0.000		
R-Squared		0.117		
Adjusted R-square		0.05		

* Significance at *p* < 0.05, ** Significance at *p* < 0.01, *** Significance at *p* < 0.001.

## Data Availability

Not applicable.
